# Compliance with the vaccination schedule in children hospitalized with pneumonia and associated factors

**DOI:** 10.11606/S1518-8787.2018052006888

**Published:** 2018-04-04

**Authors:** Amanda Tabosa Pereira da Silva, Eduardo Jorge da Fonseca Lima, Maria de Fátima Costa Caminha, Andresa Tabosa Pereira da Silva, Edil de Albuquerque Rodrigues, Carmina Silva dos Santos

**Affiliations:** I Instituto de Medicina Integral Prof. Fernando Figueira. Programa de Pós-Graduação em Cuidados Intensivos Associados à Residência. Recife, PE, Brasil; II Universidade Federal de Pernambuco. Programa de Pós-Graduação em Neuropsiquiatria e Ciências do Comportamento. Recife, PE, Brasil; III Instituto de Medicina Integral Prof. Fernando Figueira. Diretoria de Ensino. Recife, PE, Brasil; IV Instituto de Medicina Integral Prof. Fernando Figueira. Diretoria de Pesquisa. Recife, PE, Brasil; V Instituto de Medicina Integral Prof. Fernando Figueira. Programa de Pós-Graduação em Educação para Ensino em Saúde. Recife, PE, Brasil; VI Universidade Federal de Pernambuco. Programa de Pós-Graduação em Saúde da Criança e do Adolescente. Recife, PE, Brasil

**Keywords:** Child, Inpatients, Pneumonia, epidemiology, Immunization Coverage, Immunization Programs, Criança, Pacientes Internados, Pneumonia, epidemiologia, Cobertura Vacinal, Programas de Imunização

## Abstract

**OBJECTIVE:**

To verify the adequacy and factors associated with compliance with the immunization schedule (BCG, DTP-Hib, MMR, PCV-10) in children hospitalized with pneumonia at a pediatric referral hospital in Northeast Brazil.

**METHODS:**

This is a cross-sectional, descriptive study with an analytical component, with a sample of 452 children hospitalized with pneumonia at the Instituto de Medicina Integral Prof. Fernando Figueira, between 2010 and 2013. The inclusion criterion was children aged from one month to less than five years of age with proof in the immunization record. The exclusion criterion was the presence of hospital-acquired pneumonia or concomitant disease. We have evaluated the adequacy of the immunization schedule for the BCG, tetravalent, MMR, and 10-valent pneumococcal conjugate (PCV-10) vaccines. We used the chi-square test and Fisher's exact test followed by multivariate Poisson regression, estimating the crude and adjusted prevalence ratios and respective 95% confidence intervals. The variables with p < 0.20 in the univariate analysis were included in the multivariate analysis.

**RESULTS:**

There was good adequacy in the immunization schedule, except for PCV-10, which presented a percentage lower than 85%. We have observed an association between adequate compliance with the immunization schedule and education level of the mother (89.9% complete high school), sex of the child (87.2% female), age of the child (94.2% younger than six months), and breastfeeding (84.3% breastfed).

**CONCLUSIONS:**

Given the high rate of education level of the mother and the high percentage of breastfeeding, we can understand that there is a better understanding of the health of the child by the mothers studied in this study, showing the effectiveness of public policies for infant feeding. However, children did not have good adequacy of the immunization schedule of PCV-10, one of the main vaccines against pneumonia, which can be one of the main factors in the causes of hospitalization, with no influence on the classification of the severity of the disease. In this way, we emphasize that the causes of pneumonia morbidity are not associated with a single factor.

## RESUMO

**OBJETIVO:**

Verificar a adequação e os fatores associados ao cumprimento do esquema vacinal (BCG, DTP-Hib, SCR, VCP-10) em crianças internadas com pneumonia em um hospital de referência pediátrica no Nordeste do Brasil.

**MÉTODOS:**

Estudo transversal, descritivo com componente analítico, composto por 452 crianças hospitalizadas por pneumonia no Instituto de Medicina Integral Prof. Fernando Figueira, entre 2010 e 2013. Critérios de inclusão: idade de um mês a menores de cinco anos; com comprovação do cartão vacinal. Critérios de exclusão: pneumonia hospitalar ou doença de base concomitante. Avaliamos a adequação do esquema vacinal da BCG, tetravalente, tríplice viral e pneumocócica conjugada 10 valente (VPC-10). Foram utilizados os testes qui-quadrado e exato de Fisher seguidos de regressão multivariada de Poisson, estimando-se as razões de prevalência brutas, ajustadas e os respectivos intervalos de confiança de 95%. Participaram da análise multivariada as variáveis que na análise univariada apresentaram valor p < 0,20.

**RESULTADOS:**

Houve boa adequação no calendário vacinal, exceto a vacina VPC-10, que apresentou percentual inferior a 85%. Observou-se associação entre o adequado cumprimento do esquema vacinal e escolaridade materna (89,9% ensino médio completo), sexo da criança (87,2% feminino), idade da criança (94,2% menor que seis meses) e aleitamento materno (84,3% amamentaram).

**CONCLUSÕES:**

Pela elevada taxa na escolaridade materna e pelo elevado percentual de alimentação por leite materno, pode-se entender que há uma melhor compreensão no cuidado da saúde da criança pelas genitoras estudadas nesta pesquisa, apresentando a eficácia das políticas públicas de alimentação infantil. Porém, as crianças não tiveram uma boa adequação do esquema vacinal da VPC-10, uma das principais vacinas contra a pneumonia, podendo ser esse um dos principais fatores nas causas do internamento, não apresentando influência com a classificação da gravidade da doença. Enfatiza-se dessa maneira que as causas de morbidade por pneumonia não são associadas a um único fator.

## INTRODUCTION

Among the major diseases with an impact on infant mortality worldwide, acute respiratory infections (ARI), such as community-acquired pneumonia (CAP), account for approximately 19% of the causes of death in the world^5^ ^,^ ^14^. The World Health Organization (WHO) reports that most of the cases are in developing countries, and in this context Brazil is among the 15 countries with the highest incidence of CAP in children under five years of age^14^ ^,^ ^20^. The major risk factors for pneumonia are grouped under individual child conditions, incomplete immunization, and socioeconomic, demographic, and cultural factors^11^.

As a possible impact on pneumonia, the National Immunization Program (PNI) in Brazil currently offers the tuberculosis (BCG – Bacillus Calmette-Guérin), 10-valent pneumococcal conjugate (PCV-10V), tetravalent [specifically the anti-*Haemophilus influenzae* type b and anti-pertussis components (DTP-Hib)], and MMR (specifically the anti-measles component) vaccines^8^ ^,^ ^10^ ^,^ ^25^.

Despite the implantation of the vaccines in the immunization schedule and the availability to the population in the Brazilian Unified Health System (SUS), data recorded in the Information Technology Department of the SUS (DATASUS), in 2012, show that the rate of abandonment in immunization compliance by parents or guardians was high, which indicates that vaccination coverage is lower than expected, being the value equal to or above 95% considered as the ideal percentage of coverage for each vaccine^23^.

Vaccination coverage is a health indicator that reveals the amount of vaccine dispersed by basic health services, being carried out by the proportion of children who received complete immunization in relation to the number of children in the population of the same age group evaluated. The complete immunization schedule is characterized by the administration of the vaccines recommended by the PNI, with doses applied at the indicated ages (epidemiological adequacy) and with correct scheduling (immunological adequacy)^2^.

If children are not vaccinated they will be more susceptible to diseases, being pneumonia an example given its high incidence rate^6^ ^,^ ^17^. The justifications traditionally used by the guardians of the children for not completing the immunization schedule are: no immunization record, lack of time, difficulty to go to the vaccination unit (access), refusal of vaccination, “unit was closed”, lack of vaccine in the unit, contraindication, adverse event of a previous dose, among other reasons^12^ ^,^ ^17^.

Given the epidemiological importance of pneumonia and its lethality, studies aimed to prevent it need to be carried out. This study verified the adequacy and the factors associated with compliance with the immunization schedule (BCG, DTP-Hib, MMR, PCV-10) in children hospitalized with pneumonia at a pediatric referral hospital in Northeast Brazil.

## METHODS

This is a cross-sectional, descriptive study with an analytical component with 452 children hospitalized with community-acquired pneumonia at the Instituto de Medicina Integral Professor Fernando Figueira (IMIP). The collection period was from October 2010 to September 2013. The IMIP is a reference hospital in pediatrics, responsible for the hospitalization of approximately 30% of cases of pneumonia in children under five years in the State of Pernambuco, according to data obtained in DATASUS in 2012.

Inclusion criteria were children admitted to the IMIP with community-acquired pneumonia, aged between one month and five years, and who showed proof of the immunization schedule. We excluded children with hospital-acquired pneumonia or carriers of concomitant diseases, such as heart disease, chronic lung diseases, nephropathies, neuropathies, hemoglobinopathies, liver diseases, immunodeficiency, cystic fibrosis, and congenital lung malformations. Patient recruitment was performed in the emergency or pediatric emergency department, infirmaries, or the pediatric intensive care unit of the institution.

The diagnosis of pneumonia was based on the clinical and radiological criteria established by the WHO, including the classification of severity, for epidemiological studies of vaccine effectiveness^13^ ^,^ ^22^. The variables were classified into four groups: sociodemographic data of the mothers (age of the mother, complete high school, work of the mother, family income, household agglomeration, and smoking in the residence), biological data of the child (age in months, sex, birth weight, prematurity, breastfeeding), data on the classification of pneumonia (pneumonia, severe pneumonia, and very severe pneumonia), and data on the adequacy of the vaccine (BCG, DTP-Hib, MMR, and PCV-10V).

The classification of adequacy for each vaccine occurred according to the age of the child, which ranged from one to 59 months, and the schedule recommended in the Basic Childhood Immunization Schedule recommended by the Ministry of Health between 2006 and 2013. Thus, we considered as adequate the cases in which the immunization record of the child showed that a dose was applied at the indicated age and with the appropriate schedule (Box).

**Box:** Definition of vaccines investigated in children hospitalized with pneumonia at the Instituto de Medicina Integral Prof. Fernando Figueira. Pernambuco, Brazil, October/2010 to September/2013.

Vaccine	Description	Scheduling	Adequacy
BCG	The BCG vaccine is administered at birth in large maternity hospitals in Brazil since 1991^a^.	At birth, one dose	It was considered as adequate for children with a proven dose
Tetravalent (DTP-Hib)	In 2002, the tetravalent conjugate vaccine (diphtheria, tetanus, pertussis, and *Haemophilus influenzae* type b – conjugate) was implanted. In 2013, another component was added, becoming a pentavalent vaccine (diphtheria, tetanus, pertussis, hepatitis B, and *Haemophilus influenzae* type b – conjugate)^a^,^b^.	The scheduling of this vaccine remained the same regardless of the addition of the hepatitis B component. 1st dose – 2 months 2nd dose – 4 months 3rd dose – 6 months	It was considered as adequate children who were in accordance with the number of doses established by the Ministry of Health according to their age in months: 1 to < 2 months: zero dose 2 to < 4 months: 1 dose 4 to < 6 months: 2 doses 6 to < 60 months: 3 doses
MMR	In 2003, the MMR (measles, mumps, and rubella) conjugate vaccine was implanted. By 2013, another component was added, being then denominated MMRV (measles, mumps, rubella, and varicela)^a^,^c^.	There was a change in the scheduling of the vaccine: In 2003 – MMRV: 1 dose after 12 months. In 2013 – MMRV: 1st dose – 12 months MMR: 2nd dose: after 15 months	It was considered as adequate children who were in accordance with the number of doses established by the Ministry of Health according to their age in months: Before 2013: Children > 12 months should present 1 single dose of the MMR After 2013: Children > 15 months should present 2 doses, being the 1st one the MMR and the 2nd one the MMRV
10-valent pneumococcal conjugate (PCV-10)	In 2010, the PCV-10 was implemented, which requires four different schedules^a^,^d^. In 2016, there was a change in the scheduling of this vaccine, being given at 2 and 4 months (1st and 2nd dose, respectively) and booster at 12 months. However, this new scheduling does not interfere in the analysis of this research, since this modification was performed after the collection of the data.	In the first year of implantation of this vaccine, the MH stipulated four different types of vaccination schedule, determined by the age of the child, being them^a^,^d^: 2 to 6 months: 3 doses and 1 booster 7 to 11 months: 2 doses and 1 booster 10 to 13 months: 2 doses and no booster 12 to < 24 months: 1 dose and no booster	It was considered as adequate children who were in accordance with the number of doses and booster established by the MH according to their age in months: In 2010: 2 to 6 months: 4 administrations 7 to 11 months: 3 administrations 10 to 13 months: 2 administrations 12 to < 24 months: 1 administration After 2011: < 2 months: 1 dose 2 to < 4 months: 1 dose 4 to < 6 months: 2 doses 6 to < 12 months: 3 doses > 12 months: 3 doses and 1 booster

MH: Ministry of Health

a Ministério da Saúde (BR), Secretaria de Vigilância em Saúde, Departamento de Vigilância Epidemiológica. Programa Nacional de Imunizações (PNI): 40 anos. Brasília (DF); 2013 [cited 2017 Jul 17]. Available from: http://bvsms.saude.gov.br/bvs/publicacoes/programa_nacional_imunizacoes_pni40.pdf

b Ministério da Saúde (BR), Secretaria de Vigilância em Saúde, Departamento de Vigilância Epidemiológica, Coordenação Geral do Programa Nacional de Imunizações. Informe técnico da introdução da vacina pentavalente: vacina adsorvida difteria, tétano, *pertussis*, hepatite B (recombinante) e *Haemophilus influenzae* tipo b (conjugada). Brasília (DF); 2012 [cited 2017 Jul 14]. Available from: http://www.sgc.goias.gov.br/upload/arquivos/2012-06/informe-tecnico-vacina-pentavalente.pdf

c Ministério da Saúde (BR), Secretaria de Vigilância em Saúde, Departamento de Vigilância Epidemiológica, Coordenação Geral do Programa Nacional de Imunizações. Informe técnico de introdução da vacina tetra viral: vacina sarampo, caxumba, rubéola e varicela (atenuada). Brasília (DF); 2013 [cited 2017 Jul 17]. Available from: http://www.sopape.com.br/data/conteudo/arquivos/informe_tecnico_introducao_vacina_tetraviral.pdf

d Ministério da Saúde (BR), Secretaria de Vigilância em Saúde, Departamento de Vigilância Epidemiológica, Coordenação Geral do Programa Nacional de Imunizações. Informe técnico da vacina pneumocócica 10-valente (conjugada). Brasília (DF); 2010 [cited Feb 2010]. Available from: http://www.sgc.goias.gov.br/upload/links/arq_723_infotec.pdf

The data was typed in Excel 2007 in duplicate, validated in EpiInfo 3.5.2, and analyzed in Stata 12.1. The analysis of the factors associated with the adequate immunization schedule for the BCG, DTP-Hib, MMR, and PCV-10V vaccines, based on the sociodemographic variables of the mother and the biological variables of the children, was performed using multivariate Poisson regression and estimating the crude and adjusted prevalence ratios (PR) and their respective 95% confidence intervals. The variables with p < 20% in the univariate analysis were included in the multivariate analysis. In order to analyze the frequency distribution and association of the days of hospitalization, ICU stay, and evolution to discharge or death as a function of the adequate immunization schedule for the studied vaccines, we used the chi-square test and the Fisher's exact test when necessary. We considered the < 5% level of significance for all tests. The vaccines studied (BCG, DTP-Hib, MMR, and PCV-10) were considered as independent variables, and the other variables as dependent.

This study was approved by the Research Ethics Committee of the Instituto de Medicina Integral Prof. Fernando Figueira, CAAE 30928314.9.0000.5201, under Protocol 4196-14 approved on June 11, 2014, according to Resolution 466/12 of the National Health Council of the Ministry of Health of Brazil related to research with human beings.

## RESULTS

A total of 452 children hospitalized with pneumonia were studied from October 2010 to September 2013, with mean age of 17.5 months and median of 14 months. Tables 1, 2, and 3 describe the distribution of frequencies according to the adequacy of the vaccine, in relation to the sociodemographic and biological variables, pneumonia classification, length of hospitalization and intensive care unit, and evolution.

**Table 1:** Adequacy of the immunization schedule according to the sociodemographic variables of the mother and the biological variables of the children hospitalized with pneumonia at the Instituto de Medicina Integral Prof. Fernando Figueira. Pernambuco, Brazil, October/2010 to September/2013.

Variable		Specification of the results	Appropriate immunization schedule					
			Sample n = 452^a^	n (%)	Crude PR (95%CI)	p^b^	Adjusted PR (95%CI)	p^b^
Sociodemographic variables of the mother								
Age (years)						0.273		
	< 20 and 36 or more		105	83 (79.0)	1			
	20 to 35		344	289 (84.0)	1.06 (0.95–1.18)			
Complete high school						0.001		0.001
	Yes		168	151 (89.8)	1.14 (1.05–1.23)		1.15 (1.06–1.25)	
	No		270	213 (78.8)	1		1	
Work of the mother						0.669		
	Yes		119	97 (81.5)	1			
	No		329	274 (83.2)	1.02 (0.92–1.12)			
Family income (MW)						0.004		0.137
	≤ 1		275	217 (78.9)	1		1	
	> 1		177	157 (88.7)	1.12 (1.04–1.22)		1.07 (0.98–1.17)	
Agglomeration						0.279		
	Yes		118	93 (78.8)	1			
	No		322	269 (83.5)	1.06 (0.95–1.18)			
Smoking at the house						0.085		0.240
	Yes		139	108 (77.7)	1		1	
	No		311	264 (84.8)	1.09 (0.99–1.21)		1.06 (0.96–1.17)	
Biological variables of the children								
Sex						0.016		0.002
	Male		234	184 (78.6)	1		1	
	Female		218	190 (87.1)	1.11 (1.02–1.20)		1.13 (1.05–1.23)	
Birth weight (g)						0.273		
	< 2,500		47	36 (76.6)	1			
	≥ 2,500		368	309 (83.9)	1.10 (0.93–1.29)			
Prematurity						0.404		
	Yes		36	28 (77.7)	1			
	No		387	325 (83.9)	1.08 (0.90–1.29)			
Age						< 0.001		< 0.001
	< 6 months		119	112 (94.1)	1.31 (1.16–1.47)		1.35 (1.20–1.53)	
	6 to 12 months		85	72 (84.7)	1.18 (1.02–1.36)		1.21 (1.05–1.39)	
	13 to 24 months		120	98 (81.6)	1.14 (0.99–1.30)		1.15 (1.01–1.32)	
	> 24 months		128	92 (71.8)	1		1	
Breastfeeding						0.031		0.029
	Yes		414	349 (84.3)	1.35 (1.03–1.77)		1.32 (1.03–1.68)	
	No		32	20 (62.5)	1		1	

MW: minimum wage

a The sample varied because of the lack of information.

b Poisson.

**Table 2:** Distribution of frequency of compliance with the schedule for the BCG, Tetravalent, MMR, and PCV-10 vaccines according to the classification of pneumonia in children hospitalized with pneumonia at the Instituto de Medicina Integral Prof. Fernando Figueira. Pernambuco, Brazil, October/2010 to September/2013. (n = 452^a^)

Adequacy of the vaccine		Classification of pneumonia				p^b^
		Pneumonia	Severe	Very severe	Total	
		n (%)	n (%)	n (%)	n (%)	
BCG						0.146
	Yes	73 (16.3)	364 (81.4)	10 (2.2)	447 (100)	
	No	0 (0)	4 (80.0)	1 (20.0)	5 (100)	
Tetravalent						0.305
	Yes	73 (16.6)	355 (80.9)	11 (2.5)	439 (100)	
	No	0 (0)	13 (100)	0 (0)	13 (100)	
MMR						1.000
	Yes	72 (16.1)	363 (81.4)	11 (2.5)	446 (100)	
	No	1 (16.7)	5 (83.3)	0 (0)	6 (100)	
Pneumococcal^a^						0.218
	Yes	62 (16.8)	297 (80.3)	11 (3.0)	370 (100)	
	No	8 (11.59)	61 (88.4)	0 (0)	69 (100)	

BCG: Bacillus Calmette-Guérin; PCV-10: 10-valent pneumococcal conjugate vaccine

a The sample varied by analyzing only children of compatible age at the time of vaccine implantation (n = 439).

b Chi-square test and Fisher's exact test.

**Table 3:** Distribution of frequency of compliance with the schedule for the BCG, Tetravalent, MMR, and PCV-10 vaccines according to total days of hospitalization, ICU stay, and evolution to discharge or death in children hospitalized with pneumonia at the Instituto de Medicina Integral Prof. Fernando Figueira. Pernambuco, Brazil, October/2010 to September/2013. (n = 452^a^)

Adequacy of the vaccine		Time of hospitalization		Total	p^b^
		≤ 7 days	> 7 days	n (%)	
		n (%)	n (%)		
BCG					0.212
	Yes	227 (50.9)	219 (49.1)	446 (100)	
	No	1 (20.0)	4 (80.0)	5 (100)	
Tetravalent					0.576
	Yes	220 (50.2)	218 (49.7)	438 (100)	
	No	8 (61.5)	5 (38.4)	13 (100)	
MMR					1.000
	Yes	225 (50.5)	220 (49.4)	445 (100)	
	No	3 (50.0)	3 (50.0)	6 (100)	
Pneumococcal^a^					0.795
	Yes	186 (50.4)	183 (49.5)	369 (100)	
	No	36 (52.1)	33 (47.8)	69 (100)	
		ICU stay			
		Yes	No		
BCG					1.000
	Yes	17 (3.8)	430 (96.2)	447 (100)	
	No	0 (0)	5 (100)	5 (100)	
Tetravalent					1.000
	Yes	17 (3.9)	422 (96.1)	439 (100)	
	No	0 (0)	13 (100)	13 (100)	
MMR					1.000
	Yes	17 (3.8)	429 (96.2)	446 (100)	
	No	0 (0)	6 (100)	6 (100)	
Pneumococcal^a^					0.218
	Yes	15 (4.1)	355 (95.9)	370 (100)	
	No	1 (1.4)	68 (98.5)	69 (100)	
		Evolution			
		Discharge	Death		
BCG					1.000
	Yes	440 (98.4)	7 (1.6)	447 (100)	
	No	5 (100)	0 (0)	5 (100)	
Tetravalent					1.000
	Yes	432 (98.4)	7 (1.6)	439 (100)	
	No	13 (100)	0 (0)	13 (100)	
MMR					1.000
	Yes	439 (98.4)	7 (1.6)	446 (100)	
	No	6 (100)	0 (0)	6 (100)	
Pneumococcal^a^					0.603
	Yes	363 (98.1)	7 (1.9)	370 (100)	
	No	69 (100)	0 (0)	69 (100)	

BCG: Bacillus Calmette-Guérin; PCV-10: 10-valent pneumococcal conjugate vaccine; ICU: intensive care unit

a The sample varied by analyzing only children of compatible age at the time of vaccine implantation (n = 439).

b Chi-square test and Fisher's exact test.

The Figure presents the compliance of the BCG, tetravalent, MMR, and PCV-10 vaccines. The latter presented the lowest percentage in the adequacy of vaccination compliance, being less than 85%; the other vaccines showed a percentage of adequacy higher than 97%.

**Figure:**
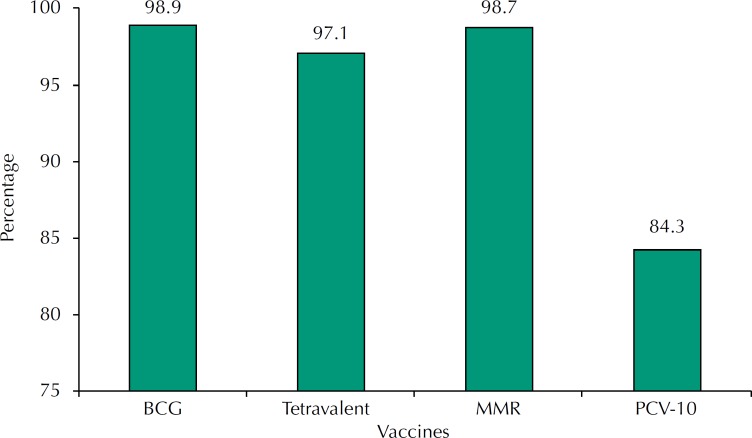
Distribution of frequency of the adequacy of the compliance with the vaccination schedule according to the specific vaccines of the children hospitalized with pneumonia at the Instituto de Medicina Integral Prof. Fernando Figueira. Pernambuco, Brazil, October/2010 to September/2013. BCG: Bacillus Calmette-Guérin; PCV-10: 10-valent pneumococcal conjugate vaccine

Table 1 shows the analysis between compliance with the immunization schedule and the sociodemographic variables of the mother and the biological variables of the children hospitalized with pneumonia at IMIP. Regarding the variables verified in the bivariate analysis, we highlight that complete high school, family income greater than one minimum wage, female sex, age of the children, and breastfeeding had a statistically significant association with compliance with the immunization schedule. To evaluate the independent effect of each of these variables, we adjusted the multivariate Poisson regression model, in which only the education level of the mother, female sex, age of the children, and breastfeeding remained significant.

Table 2 shows the frequency distribution and association of the classification of pneumonia according to the adequate immunization schedule for the BCG, tetravalent, MMR, and PCV-10 vaccines of children hospitalized with pneumonia at IMIP. We observed no statistically significant difference.

Table 3 shows the frequency distribution of the compliance with the immunization schedule according to length of hospitalization, ICU indication, and final outcome of the children hospitalized with pneumonia at IMIP. We also observed no association between these variables and the compliance with the immunization schedule.

## DISCUSSION

Pneumonias are an important cause of mortality worldwide and in Brazil, thus the global action plan for the prevention and control of pneumonia (GAPP) highlights the importance of vaccines as a protective factor for the health of children. It reinforces that the use of the BCG, combined with the pertussis and Hib components, MMR, and PCV-10 vaccines is related to the reduction of disease incidence^1^ ^,^ ^9^ ^,^ ^24^.

In this study, we observed a high percentage of adequacy for the BCG, tetravalent, and MMR vaccines; however, the adequacy percentage for PCV-10 was lower than that recommended by the Ministry of Health^23^. Although we analyzed the immunization records of the hospitalized children, the coverage results were similar to those found in other populations of children in Brazil^15^.

The percentage of adequacy observed for the BCG, tetravalent, and MMR vaccine can be explained by the time of their implantation in the Brazilian immunization schedule. The BCG vaccine has been available since the first implementation of the immunization record by the Ministry of Health (MH) in 1973, similarly to the monovalent vaccine against measles^a^. The tetravalent and MMR conjugate vaccines were added to the immunization schedule in 2002 and 2003, respectively.

On the other hand, the time of implantation of the PCV-10 in the immunization schedule is inferior to the vaccines previously mentioned. In this way, we can conjecture the lower frequency in the percentage of vaccine coverage found. This information corroborates other studies, which attribute the low adherence to the compliance with the schedule to the recent implantation of the pneumococcal vaccine in the SUS^12^ ^,^ ^a^.

Regarding the association between compliance with the immunization schedule and the variables of the mother studied, we observed a statistically significant difference between the compliance with the schedule and the age of the mother, in which adequacy was higher in children of mothers who had completed high school. Studies have emphasized the relationship between low education level of the mother and non-compliance with the immunization schedule, especially in developing countries. Thus, women with higher education level have greater possibilities of access to health information and disease prevention and provide better care to their child^1^ ^,^ ^19^.

Regarding the age of the mother, although this variable was not statistically significant in relation to compliance with the immunization schedule, we observed a higher percentage among children of mothers aged between 20 and 35 years in relation to the adequate immunization schedule for the age. Studies show that care directed to children as a result of the immunization schedule, feeding, hygiene, education, and health are usually linked to some factors, such as: age of the mother between 20 and 35 years, education level of the mother greater than or equal to eight years, and income higher than one minimum wage. In this way, it is expected that families with these characteristics take greater care of the health of their child, which was confirmed in our study^1^ ^,^ ^4^ ^,^ ^20^.

Moreover, according to the socioeconomic variables, in terms of family income, more than half of the children lived with families that had income below one minimum wage, a characteristic result of developing countries^3^ ^,^ ^19^; however, there was no association with compliance with the immunization schedule in the children evaluated in our study. Evidence shows that families with poor socioeconomic background present a 2.4 times greater risk of hospitalization, which emphasizes that the socioeconomic level of the population influences health care, since access and search for information are more restricted^3^ ^,^ ^19^.

Regarding the biological variables of the child, there was an association between sex and compliance with the immunization schedule, with higher percentage of vaccine coverage for girls, although the population studied was predominantly male. The sex of the child as a risk factor for pneumonia is not fully understood and there is still no consensus in the literature^5^ ^,^ ^18^. It is known that males are more susceptible to low respiratory infections and that females are more resistant to infections, which is explained by the better immunity response in girls^5^ ^,^ ^18^.

According to the age of the child, there was an association with compliance with the immunization schedule, in which there was a decrease in the adequacy percentage as the age group increased. This can be conjectured considering that children have more appointments in the first year of life, especially in the first semester, which monitor child development, allowing greater access to health services and, consequently, greater adherence to the immunization schedule^16^.

We also observed an association between the adequacy of the schedule in relation to breastfeeding, since the percentage of adequacy was higher among children who breastfed. This can be explained by the fact that during the first months of life the child is breastfeeding; the recommendation of the MH is breastfeeding for two years. The exclusive practice is for six months of life, a period on which all the efforts of public health policy services are focused. The scientific evidence states that the association between breastfeeding and compliance with the immunization schedule reinforces the protection against diseases in children^4^ ^,^ ^21^ ^,^ ^24^.

In addition, children who are not breastfed are 61 times more likely to be hospitalized with pneumonia when compared to children who were exclusively breastfed for six months^24^. A study carried out in Sweden indicates that children with exclusive breastfeeding for 90 days or more who had the Hib and pneumococcal vaccine, concurrently, obtained better serological protection when compared to children who were breastfed for a few days^21^. In this way, its corroborates that these practices carried out concomitantly, exclusive breastfeeding for six months and the fulfillment of the immunization schedule, could give a greater protection to children in this age group^7^ ^,^ ^21^.

Regarding the classification of pneumonia, the hospitalization period, and the indication of ICU of the hospitalized children, we observed no association with compliance with the immunization schedule, although most of the children presented adequate scheduling. Even in this study, although we observed an association between compliance with the immunization schedule and other variables such as education level of the mother, sex, age of the child, and breastfeeding, we understand that the health-disease process is linked to a series of multifactorial events and not exclusively to the adequacy of the immunization schedule. However, the PCV-10 is an important and specific factor for protection against pneumonia, decreasing vulnerability and promoting the health of the population, especially children.

As this study only intended to verify compliance with the immunization schedule, we could not define the immunological status of the child after vaccination, which is a limitation of the study. Children with pneumonia, regardless of classification, might even have an updated but incomplete scheduling, which would leave them still vulnerable. Similarly, children who spent more days hospitalized or who needed hospitalization in the ICU also did not have their serological situation verified in relation to the vaccines received.

For public policies, this study presents, in a general way, the efficiency and effectiveness in infant feeding and in the investment of vaccination campaigns, as we observed an adequacy for these main highlights. Despite the data evaluated, new studies need to be carried out to evaluate public health programs and the immunological status of the vaccinated children.

## Footnotes

a Ministério da Saúde (BR), Secretaria de Vigilância em Saúde, Departamento de Vigilância Epidemiológica, Coordenação Geral do Programa Nacional de Imunizações. Informe técnico da introdução da vacina pentavalente: vacina adsorvida difteria, tétano, *pertussis*, hepatite B (recombinante) e *Haemophilus influenzae* tipo b (conjugada). Brasília (DF); 2012 [cited 2017 Jul 14]. Available from: http://www.sgc.goias.gov.br/upload/arquivos/2012-06/informe-tecnico-vacina-pentavalente.pdf
